# A Randomized Controlled Trial to Investigate the Effectiveness of the Prevention of Aspiration Pneumonia Using Recommendations for Swallowing Care Guided by Ultrasound Examination

**DOI:** 10.3390/healthcare6010015

**Published:** 2018-02-12

**Authors:** Yuka Miura, Gojiro Nakagami, Koichi Yabunaka, Haruka Tohara, Hiroshi Noguchi, Taketoshi Mori, Hiromi Sanada

**Affiliations:** 1 Department of Gerontological Nursing/Wound Care Management, Graduate School of Medicine, The University of Tokyo, 7-3-1 Hongo, Bunkyo-ku, Tokyo 113-0033, Japan; yukam-tky@umin.ac.jp (Y.M.); gojiron-tky@umin.ac.jp (G.N.); 2 Global Nursing Research Center, Graduate School of Medicine, The University of Tokyo, 7-3-1 Hongo, Bunkyo-ku, Tokyo 113-0033, Japan; kyabunaka-tky@umin.ac.jp; 3 Department of Imaging Nursing Science, Graduate School of Medicine, The University of Tokyo, 7-3-1 Hongo, Bunkyo-ku, Tokyo 113-0033, Japan; 4 Gerodontology and Oral Rehabilitation, Department of Gerontology and Gerodontology, Graduate School of Medical and Dental Sciences, Tokyo Medical and Dental University, 1-5-45 Yushima, Bunkyo-ku, Tokyo 113-8549, Japan; haruka-t@rd5.so-net.ne.jp; 5 Department of Life Support Technology (Molten), Graduate School of Medicine, The University of Tokyo, 7-3-1 Hongo, Bunkyo-ku, Tokyo 113-0033, Japan; hnogu-tky@umin.ac.jp (H.N.); tmoriics-tky@umin.ac.jp (T.M.)

**Keywords:** aspiration pneumonia, deglutition disorders, swallowing care, ultrasound

## Abstract

Prevention for aspiration pneumonia requires assessment of aspiration and adequate swallowing care. This randomized controlled trial aimed to investigate the effectiveness of ultrasound examination and recommendations for swallowing care for the reduction of aspiration and pharyngeal post-swallow residue as compared with standard swallowing care. Twenty-three participants were randomized to the intervention group and 23 to the control group. The intervention consisted of four ultrasound examinations during mealtimes and recommendations for swallowing care every 2 weeks during an 8 week period. No recommendations concerning swallowing care based on ultrasound examinations were provided to the control group. The frequency of aspiration or residue was defined as x/y × 100% when aspiration or residue were detected x times from y times concerning the total ultrasound measurements. The proportion of the residents with reduced frequency of aspiration which was detected by ultrasonography at eight weeks were 4.3% in the intervention group and 0% in the control group. The median reduction in the frequency of aspiration and residue in the intervention group was 31%, and that in the control group was 11%. In conclusion, swallowing care guided by frequent ultrasound examinations during mealtimes had a trend of reducing the frequency of aspiration and residue during an 8-week period in individuals relative to standard swallowing care alone.

## 1. Introduction

Prevention of aspiration pneumonia is one of the major concern in health care for elderly people because of its high mortality and recurrence [1,2]. The estimated annual number of cases of aspiration pneumonia in Japan, where is the most aged society in the world, were 630,000 in 2013 [3]. Aspiration pneumonia is caused from oropharyngeal microorganisms contained in liquids, foods, and saliva that flow into airway, which is defined as aspiration [4]. The pharyngeal post-swallow residue is the presence of food or liquid in the hypopharynx that is not entirely eliminated by swallowing [5]. The accumulated pharyngeal post-swallow residue sometimes flows into the airway without cough reflex in people with dysphagia. Therefore, detecting both aspiration and pharyngeal post-swallow residue, and providing swallowing care to reduce risk of aspiration are necessary for prevention of aspiration pneumonia. Videofluoroscopic swallowing study (VFSS) and fiberoptic endoscopic evaluation of swallowing (FEES) are considered gold standards to detect aspiration and pharyngeal post-swallow residue. VFSS exposes participants to ionizing radiation, and FEES requires insertion of an endoscope into the participant’s nose. Therefore, these invasive assessment methods are not appropriate to use as a daily assessment during mealtime. Daily assessments for swallowing problems are important because the swallowing conditions of elderly people will be affected by the kinds of food and liquid they ingest and by the small changes in their physical and cognitive status [6,7,8].

Previous studies succeeded in visualizing aspirated boluses in the trachea during swallowing by ultrasound (US) [9,10]. Since the US has the advantages of noninvasiveness and portability as an imaging method, it is useful as a daily assessment tool for swallowing problems. Aspirated boluses during swallowing appeared as a hyperechoic line along the tracheal wall that passed through the vocal folds in the cases in which aspiration was detected by VFSS and FEES. The sensitivity of aspiration detection during swallowing was 64%, and the specificity was 84%. To increase the sensitivity and specificity, an image processing method based on the movement of the aspirated boluses, which was different from the movement of the surrounding tissue caused by swallowing, was applied. As expected, this method assisted detection of the aspirated boluses from complex US images, improving the sensitivity and specificity to 91% and 94%, respectively [11]. Pharyngeal post-swallow residue was also visualized by US as a misty hyperechoic object above the vocal folds after swallowing [12]. The sensitivity and specificity of US examination for detecting pharyngeal post-swallow residue were 67% and 75%, respectively.

Some previous studies showed that frequent use of VFSS or FEES contributed to the lower incidence of aspiration pneumonia for patients after stroke [13,14]. Advice on safe swallowing and dietary modification were conducted based on VFSS, FEES, and clinical observation. Daily swallowing assessment and appropriate swallowing care will be crucial in preventing aspiration pneumonia; however, swallowing examination involving the frequent use of imaging methods with VFSS and FEES was sometimes unfeasible because of their invasiveness. Application of less invasive US examinations during mealtimes for the detection of aspiration and pharyngeal post-swallow residue are considered to be more feasible than application of VFSS and FEES. We hypothesized that frequent swallowing examinations using the US method with recommendations for swallowing care reduces aspiration and pharyngeal residue, which are risk factors for aspiration pneumonia. The aim of this study was to investigate the effectiveness of US examination and recommendations for swallowing care for the reduction of aspiration and pharyngeal post-swallow residue as compared with standard swallowing care.

## 2. Materials and Methods

### 2.1. Study Design and Participants

The study was a prospective open randomized-controlled, parallel design trial, with one-to one allocation ratio. It is registered with the UMIN Clinical Trials Registry, number UMIN000016002. The full trial protocol is available at https://upload.umin.ac.jp/cgi-open-bin/ctr/ctr.cgi?function=brows&action=brows&recptno=R000018600&type=summary&language=E. The study has been conducted in accordance with the CONSORT Statement [15].

The study was conducted in a special elderly nursing home in the Tokyo metropolitan area, from December 2014 to September 2015. Recruitment of the participants was conducted from December 2014 to June 2015. Residents received written and oral explanations regarding the study. If a resident was assessed who had difficulty understanding the explanation of the study given by the ward manager, written or oral explanation was provided for their proxies via mail or during their visitation. All residents who underwent oral feeding were assessed for eligibility. Residents that did not provide consent, or consent was not given by their proxies, were excluded from the study. The study protocol was approved by the Ethics Committee of the Graduate School of Medicine, The University of Tokyo (#10707). Written informed consent was obtained from all residents or their proxies.

### 2.2. Randomization and Blinding

A stratified randomization was introduced in this study. The risk of aspiration pneumonia is related to the severity of swallowing disorders [16]; therefore, if many of the individuals with severe swallowing disorders are allocated to either group, the outcome of that group will be biased. To reduce such bias, stratified randomization method was adopted. Participants who satisfied at least the following three criteria were labeled as the “intensive-care group”. The other participants were labeled as the “none intensive-care group”. The criteria were as follows: (1) underwent FEES within the past 6 months or had a scheduled FEES during the study period; (2) underwent diet modification to mousse type food or mixer type food; (3) had already been introduced to alternate swallowing based on FEES or clinical observation. The intensive-care group was considered to have more severe swallowing disorders than the none-intensive-care group.

Participants were randomly assigned to the intervention group or the control group based on a randomization procedure using a single computer-generated random numbers list, held in the research office remote from the study environment. After informed consent was provided, a researcher assessed whether a participant should be included in the intensive-care group or none-intensive-care group. Then the information was provided to another researcher who performed the randomization. Other characteristics of the participants were not provided to the researcher to avoid bias involving the randomization. Blinding was not possible for participants, health care providers, and the researcher who performed US examinations; this was because it was clear to these groups that the participants underwent US examination during mealtimes.

### 2.3. Algorithm of Recommendations for Swallowing Care

**Figure 1:**
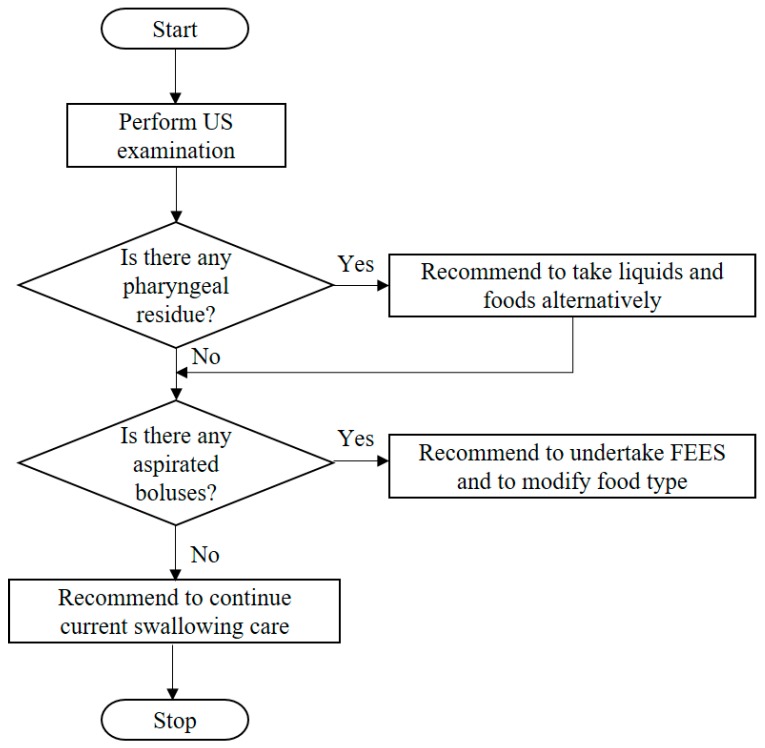
Algorithm of recommendations for swallowing care.

An algorithm regarding recommendations for swallowing care was introduced in this study (Figure 1). When pharyngeal post-swallow residue was detected during US examination at mealtimes, alternate swallowing was recommended. Alternate swallowing is one of the common strategies that are used to reduce pharyngeal post-swallow residue, because alternate solids and liquids can wash down the remaining boluses from the pharynx [17].

When aspiration was detected during US examination at mealtimes, modification of the food type and FEES were recommended. Based on previous studies, modification of the viscosity of food is one of the most effective intervention approaches in preventing aspiration pneumonia [18,19]. Aspiration involves the unintentional flow of foods or liquids boluses into the trachea that occurs in individuals with an impaired swallowing reflex. It is more difficult for these individuals to safely swallow a chopped meal or a low viscosity liquid because it scatters in the oral and pharyngeal cavity. Increasing the viscosity of the food and liquid improves bolus formation before swallowing so the level of aspiration can be reduced [20]. FEES can visualize structural abnormalities of the pharynx and larynx, which sometimes cause swallowing disorders. A method for the detection of structural disorders using US examination has not yet been established; thus, FEES was added to the recommendations for cases with aspiration to investigate the causes of aspiration. They undertook the recommendation of additional modification of food type or positioning for safe swallowing after FEES was performed. In the cases in whom pharyngeal post-swallow residue and aspiration were detected, alternate swallowing, modification of the food type and FEES were recommended. US operator recommended continuation of usual care for the participants in whom aspiration and residue were not detected.

### 2.4. Intervention

The intervention consisted of four times US examinations during mealtimes and recommendations for swallowing care based on the algorithm, every 2 weeks during an 8 weeks period. FEES for providing preventive swallowing care for aspiration pneumonia was performed once monthly for some participants. Therefore, a study period that was >1 month was required to compare the effectiveness of the intervention to usual swallowing care. The participants in whom aspiration or residue from US examination was detected, and who did not undergo a change in swallowing care, developed aspiration pneumonia in <8 weeks in the preliminary study in this nursing home; therefore, the follow-up period for investigation of the effectiveness of the intervention was 8 weeks. US examinations during mealtimes were performed by a research nurse using the same equipment as that detailed in previous studies. Baseline and follow-up examinations were performed twice over a 2 days period to avoid random error regarding the outcome, as a result of the participants’ condition and preferences concerning the contents of the meal. Because the US examinations needed to detect constant aspiration and pharyngeal post-swallow residue during mealtimes that were related to the development of aspiration pneumonia, the occurrence of a single aspiration or residue during a 2 days period was regarded as an error. If more than two detections of aspiration or residue were obtained, it was considered that the participant had a swallowing disorder that would cause aspiration pneumonia. The US examination consisted of multiple measurements involving different viscosities of food and liquid, and the presence or absence of aspiration and pharyngeal post-swallow residue at each measurement were recorded. One US examination basically consisted of nine measurements (staple diet, three measurements; side dishes, three measurements; liquids, three measurements) unless there were any difficulties encountered in collecting the images. One measurement involved a US movie of swallowing over a period of 10 s; the US examination during mealtime was completed within 10 min. Aspiration during swallowing on a US image was interpreted as the passage of a hyperechoic object through the vocal folds along the tracheal wall, involving movement that differed from that of the surrounding tissue [9]. Pharyngeal post-swallow residue was defined as the remaining hyperechoic misty area above the vocal folds after swallowing [12]. Recommendations to health care providers regarding swallowing care using US images were provided by a US operator through a full-time dietitian.

No recommendations concerning swallowing care were provided to the control group by a US operator. They undertook US examinations at the baseline and follow-up regarding the evaluation of the outcome. The swallowing care conducted in the control group included recommendation for alternate swallowing, modification of the food type. The difference from the intervention group was that these recommendations were based on the usual observations and standard examinations including scheduled FEES without using US examinations.

### 2.5. Outcome Measures

The primary outcome was the proportion of the residents with reduced frequency of aspiration which was detected by ultrasonography at eight weeks. The frequency of aspiration or residue was defined as x/y × 100% when aspiration or residue were detected x times from y times concerning the total US measurements. The secondary outcomes were the changes in swallowing care in accordance with the algorithm and the incidence of aspiration pneumonia. The changes in swallowing care were unscheduled FEES, the introduction of alternate swallowing, and modification of food type. These changes were evaluated every week by a dietitian. Aspiration pneumonia was diagnosed by physicians at the hospitals where participants were admitted for treatment of pneumonia. These physicians were blinded to the results of US examinations. Participants were diagnosed with aspiration pneumonia if aspiration or aspirated bolus, or swallowing disorders were observed [21]. Data regarding age, sex, body mass index, and experience of VFSS and FEES after admission to the nursing home were collected from the medical records.

### 2.6. Statistical Analyses

Calculation of the study sample size was based on the preliminary investigation finding that three participants out of the eight (38%) showed a reduction in the frequency of aspiration in this nursing home. Consequently, the calculation applied 40% of the reduction of the frequency of aspiration in the intervention group, and 10% of the reduction of the frequency of aspiration in the control group, over a period of 2 months for the estimation of sample size. To achieve 80% power at the 5% (two-tailed) significance level for the reliable identification of treatment effect, the sample size required was estimated to be 32 in each group. Assuming a 10% drop out rate from the study in each group, the required sample size needed was 72 in total.

Participants who undertook baseline US examinations were included into the analyses. Participants without baseline US examinations were excluded from the analyses. They were categorized into two groups (with dysphagia and without dysphagia) based on the frequency of aspiration and residue at the baseline US examination, to confirm the equality of random allocation in terms of the presence of swallowing disorders. Residents with dysphagia group was defined as the individuals whose baseline numbers of aspirations or residue were more than twice. Residents without dysphagia group was defined as the individuals whose baseline numbers of aspirations and residues were <1. Then the outcomes of participants were investigated based on the follow-up US examinations. For the residents with dysphagia, when the frequency of aspiration and residue were reduced relative to the baseline US examinations, the participant was regarded as “reduced”, and the remaining participants were regarded as “increased”. For the residents without dysphagia group, when the number of aspirations and residues were ≤1, the participant was regarded as “maintained”, and the remaining participants were regarded as “increased”. Where there was a reduction, the differences in the frequency of aspiration and residue from baseline to follow-up were calculated for each participant. For the participants whose evaluation of the comparison was inconsistent between aspiration and residue, only those who exhibited a reduction in both aspiration and residue were defined as reduced and the others were defined as increased or maintained.

The Wilcoxon rank sum test was used for the evaluation of continuous variables and Fisher’s exact test or chi-square test were used for categorical variables. Differences in the Kaplan–Meier curves were analyzed using the log rank test. All analyses were conducted using STATA, version 14 (STATA Corp., College Station, TX, USA).

## 3. Results

**Figure 2:**
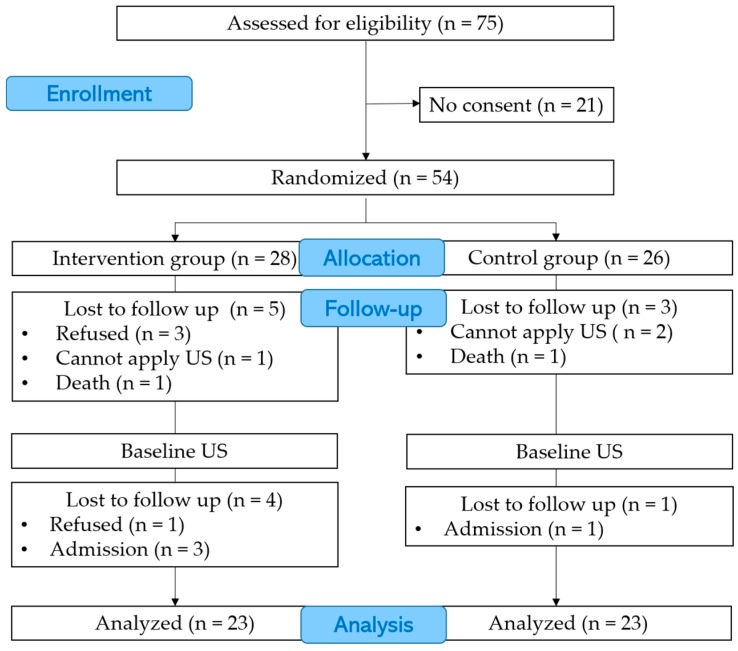
Participants Consort flow diagram. US: Ultrasound.

Seventy-five residents in total, who were capable of oral feeding in a special elderly nursing home, were assessed for eligibility before recruitment to this study. Written informed consent was obtained from 54 (72%) of the 75 eligible residents or their proxies between 19 December 2014 and 30 June 2015 (Figure 2). Follow-up was continued until 3 September 2015. Twenty-eight participants were randomized to the intervention group and 26 to the control group. In the intervention group, nine participants could not complete the 8-week follow-up period. Prior to the baseline US examinations, five participants withdrew from the study; three participants declined to join the study, one participant died, and in one participant it was judged that the attachment of the transducer was impossible because of the presence of a severe cavity in the cervical area. After the baseline US examinations, one participant refused to continue with the study, and three participants were hospitalized. In the control group, three participants could not complete follow-up. In two participants, it was considered that the attachment of the transducers was impossible because of a severely humped back or the excessive thickness of fat in the cervical area; one participant died before the baseline US examination. One participant was hospitalized after the baseline US examination. There were no cases where interruption of mealtime was observed. Twenty-three participants in the intervention and control group who undertook baseline US examination were analyzed.

Table 1 details the baseline characteristics of the participants. The intervention group included six participants and the control group included five participants allocated to the intensive-care group. The score of the Charlson Comorbidity Index was significantly higher in the control group than in the intervention group (*p* = 0.042). There were no significant differences in characteristics between the two groups including age, sex, body mass index, history of aspiration pneumonia, types of meal ingested, and the number of participants in whom aspiration and residue were detected at the baseline US examination.

The proportion of the residents with reduced frequency of aspiration which was detected by ultrasonography at eight weeks were 4.3% in the intervention group and 0% in the control group. In terms of the frequency of aspiration and residue at the follow-up US examinations in the residents with dysphagia group, two of the four participants exhibited a reduction in the frequency in the intervention group (Table 2). Three of the nine participants exhibited a reduction in the frequency in the control group.

Table 3 and Table 4 show the changes in the frequency of aspiration and residue between baseline and follow-up US examinations. The numerator indicates the numerical values of aspiration or residue detected by US examination and the denominator indicates the total values of US examination in the “baseline” and “follow-up” items. In the intervention group, all of the participants with aspiration or residue at the baseline examination showed a reduction in the frequency of the aspiration and residue at the follow-up US examination (Table 3). One participant with aspiration and one participant with residue at the baseline did not undertake follow-up US examination after 8 weeks because of admission. There were five participants in whom aspirations or residues were not detected at the baseline US examinations; they were detected at the follow-up US examinations. Any participants in the control group with aspiration at the baseline examinations did not show a reduction in the frequency of the aspiration (Table 4). Three of the participants in the control group, in whom residue was detected at the baseline examinations, exhibited a reduction in the frequency of residue. One participant with residue at the baseline did not undertake follow-up US examination after 8 weeks because of admission. Among those participants who showed a reduction in the frequency of aspiration or residue, the difference in baseline and follow-up frequency of aspiration or residue tended to be higher in the intervention group than in the control group. The median reduction in the frequency of aspiration and residue in the intervention group was 31%, and that in the control group was 11%.

In the intervention group, one participant underwent FEES and one participant underwent alternate swallowing based on the recommendations for swallowing care. Both showed a reduction in aspiration and residue after 8 weeks. One participant in the control group received a modification of food type even though there was no recommendation for swallowing care by the US operator. She did not exhibit a reduction in aspiration and residue after 8 weeks. Any scheduled FEES was not performed for participants in the intervention group or in the control group.

Two of the 23 (8.7%) participants allocated to the intervention group developed aspiration pneumonia. One of the 23 (4.3%) participants allocated to the control group developed aspiration pneumonia. There was no significant difference between the intervention and control groups regarding the time required to develop aspiration pneumonia (F = 0.450; *p* = 0.513).

## 4. Discussion

To the best of our knowledge, this is the first randomized controlled trial that has investigated the effectiveness of swallowing care guided by US examination during mealtimes as compared with standard care. The new finding was that swallowing care guided by US examination effectively reduced the frequency of aspiration and residue.

The results suggested that swallowing care guided by US examination during mealtimes effectively reduced the frequency of aspiration and pharyngeal post-swallow residue in individuals with dysphagia. Two participants out of the four in the residents with dysphagia experienced a reduction in the frequency of aspiration and pharyngeal post-swallow residue in the intervention group (Table 2). Moreover, this reduction in frequency was relatively higher in the intervention group than in the control group (Table 3 and Table 4). This finding is consistent with that of previous studies [13,14], where participants who undertook frequent VFSS or FEES were effectively prevented from developing aspiration pneumonia. The participants with dysphagia in the intervention group had not undertaken any swallowing assessments before the study, such as VFSS or FEES; therefore, there was a possibility that their dysphagia could have become worse, and that they could have developed aspiration pneumonia if they had not undertaken US examination during mealtimes.

Five participants in the intervention group experienced an increase in the frequency of aspiration or residue even though they did not show aspiration or residue at the baseline examination. Based on an algorithm for swallowing care guided by US examinations every 2 weeks, the US operator recommended continuation of usual care for the participants in whom aspiration and residue were not detected (Figure 1). The increased frequency of aspiration or residue in the intervention group indicated that the frequency of the US examinations every 2 weeks was insufficient, because there was a possibility that some deterioration in swallowing ability occurred during the 2 weeks period. The frequency of the US examination during mealtimes could be increased to detect changes in swallowing ability for more effective prevention of aspiration pneumonia.

For clinical implementation, noninvasive US examination during mealtimes will be effective in reducing the frequency of aspiration and pharyngeal post-swallow residue without causing harm. In this study, no adverse events including the interruption of mealtimes were observed in both the intervention and control groups. Nineteen (82.6%) participants were taking modified food and eight (34.8%) required some assistance for eating in the intervention group who undertook US examination during mealtimes (every 2 weeks over an 8-week period). Nineteen of the 23 (82.6%) participants in the intervention group completed the 8-week follow-up period, indicating that the frequency and duration of the US examination were generally acceptable for the participants and care staff. Two participants were excluded from this study because the transducer could not be fully attached. They had a severe cavity or excess fat in the scan area, which prevented appropriate attachment of the transducer. These participants might have had severe dysphagia caused by pharyngeal muscle atrophy [22,23]. If US operators detect participants with insufficient attachment of the transducer, the recommendation of other imaging examinations for the detection of swallowing problems for early intervention will be needed.

The strength of this study was that all residents in a special elderly nursing home were recruited. Forty-six (61%) participants of 75 accepted an invitation to join the study. In terms of generalizability, this facility is a typical nursing home in Japan. The mean age of the participants was ≥80 years; approximately 80% were women, and most undertook some food modifications [24,25]. The finding that swallowing care guided by frequent US examination contributed to a reduction in the frequency of aspiration and pharyngeal-residue may be applied to other nursing homes in Japan.

The study limitation was that an imbalance concerning the distribution of presence of dysphagia between the intervention and the control group occurred as a result of randomization. The presence of dysphagia detected at the baseline US examination was relatively higher in the control group than the intervention one (not significant). We used stratified randomization based on the application of swallowing care, which was considered to indicate the presence of dysphagia; however, this approach was not sufficient. In the next study we should use stratified randomization based on the result of baseline US examination. The introduction of swallowing assessment for the evaluation of aspiration and pharyngeal post-swallow residue before randomization might be a better approach in avoiding an imbalance in distribution. However, it was difficult to perform US examination before randomization for all participants because of the lack of a sufficient number of US operators. The application of other imaging assessments such as VFSS and FEES for all participants was ethically difficult. Future studies will be required to establish a training method for US examination during mealtimes, to evaluate the capability of this method among clinicians, and to investigate its effectiveness in preventing aspiration pneumonia using a large sample size.

## 5. Conclusions

In conclusion, swallowing care guided by frequent US examinations during mealtimes had a trend of reducing the frequency of aspiration and residue during an 8-week period in individuals with dysphagia relative to standard swallowing care alone. Further studies are required to investigate the effectiveness in reducing frequency of aspiration and residue with a large sample size.

## Figures and Tables

**Table 1:** Baseline characteristics of the participants.

	Intervention Group	Control Group	*p* Value
	(*n* = 23)	(*n* = 23)	
Age (years), median (Q1, Q3)	87 (82, 92)	85 (77, 92)	0.767 ^†^
Females, *n* (%)	20 (87.0)	20 (87.0)	1.000 ^‡^
Cerebrovascular disease, *n* (%)	7 (30.4)	11 (47.8)	0.227 ^§^
History of aspiration pneumonia, *n* (%)	5 (21.7)	1 (4.35)	0.187 ^‡^
Body mass index, median (Q1, Q3)	20.9 (18.8, 23.8)	19.3 (18.0, 21.3)	0.114 ^†^
Charlson Comorbidity Index, median (Q1, Q3)	1 (1, 2)	2 (1, 3)	0.042 ^†^
Underwent FEES or VFSS, *n* (%)	5 (21.7)	3 (13.0)	0.700 ^‡^
Type of food			0.711 ^‡^
Regular, *n* (%)	4 (17.4)	4 (17.4)	
Chopped, *n* (%)	5 (21.7)	2 (8.70)	
Soft, *n* (%)	10 (43.5)	13 (56.5)	
Mousse, *n* (%)	3 (13.0)	4 (17.4)	
Other types, *n* (%)	1 (4.35)	0 (0.00)	
Required assistance for eating, *n* (%)	8 (34.8)	7 (30.4)	0.753 ^§^
Intensive-care group, *n* (%)	6 (26.1)	5 (21.7)	0.730 ^§^
Aspiration detected by US, *n* (%)	2 (8.70)	3 (13.0)	1.000 ^‡^
Residue detected by US, *n* (%)	3 (13.0)	7 (30.4)	0.284 ^‡^
Presence of dysphagia			
With dysphagia, *n* (%)	4 (17.4)	9 (39.1)	0.189 ^‡^
Without dysphagia, *n* (%)	19 (82.6)	14 (60.9)	

FEES: fiberoptic endoscopic evaluation of swallowing; US: ultrasound; VFSS: videofluoroscopic swallowing study; Q1: first quartile; Q3: third quartile. ^†^ Wilcoxon rank sum test; ^‡^ Fisher’s exact test; ^§^ Chi-square test.

**Table 2:** Distribution of the presence of dysphagia and follow-up results.

Presence of Dysphagia	Intervention Group (*n* = 23)			Control Group (*n* = 23)		
	Baseline	Follow-Up after 8 Weeks *		Baseline	Follow-Up after 8 Weeks *	
With dysphagia, *n* (%)	4 (17.4)	Reduced	2	9 (39.1)	Reduced	3
		Increased	0		Increased	5
Without dysphagia, *n* (%)	19 (82.6)	Maintained	12	14 (60.9)	Maintained	12
		Increased	5		Increased	2

* Only participants who undertook the follow-up ultrasound examinations are shown in the table.

**Table 3:** Changes in the frequency of aspiration and residue in the intervention group.

US Results	ID	Baseline	Follow-Up after 8 Weeks	Comparison	* Difference (%)
Aspiration	19AL	10/18	4/16	Reduced	31
Residue	19AL	14/18	2/16	Reduced	65
Residue	17AL	9/18	4/18	Reduced	28

One examination basically contains nine measurements. Baseline and follow-up examination were repeated twice over 2 days. Participants in whom aspiration or residue were detected more than twice in one examination were included in this table. Participants whose frequency of aspiration and residue were reduced at follow-up relative to baseline were labeled as “Reduced”. * For the participants who were labeled as Reduced, the differences in the frequency at baseline and follow-up were calculated.

**Table 4:** Changes in the frequency of aspiration and residue in the control group.

US Results	ID	Baseline	Follow-Up after 8 weeks	Comparison	* Difference (%)
Aspiration	8BL	3/18	3/9	Increased	–
Aspiration	7BH	4/18	3/13	Increased	–
Aspiration	36BL	2/15	3/18	Increased	–
Residue	36BL	2/15	2/18	Increased	–
Residue	2BL	2/18	0/18	Reduced	11
Residue	4BL	2/18	0/18	Reduced	11
Residue	11BL	2/18	0/18	Reduced	11
Residue	21BL	2/18	2/12	Increased	–
Residue	39BL	2/10	6/18	Increased	–

One examination basically consists of nine measurements. Baseline and follow-up examination were repeated twice over 2 days. Participants in whom aspiration or residue were detected more than twice in one examination were included in this table. Participants whose frequency of aspiration and residue were reduced at follow-up relative to baseline were labeled as “Reduced”, and the remaining participant as “Increased”. * For the participants who were labeled as Reduced, the differences in the frequency at baseline and follow-up were calculated.
